# Serotonin-Related Gene Variants in Patients with Irritable Bowel Syndrome and Depressive or Anxiety Disorders

**DOI:** 10.1155/2017/4290430

**Published:** 2017-08-16

**Authors:** Magdalena Grzesiak, Jan Aleksander Beszłej, Ewa Waszczuk, Marcin Szechiński, Monika Szewczuk-Bogusławska, Dorota Frydecka, Tadeusz Dobosz, Anna Jonkisz, Arleta Lebioda, Małgorzata Małodobra, Agata Mulak

**Affiliations:** ^1^Department of Psychiatry, Wroclaw Medical University, Wroclaw, Poland; ^2^Department of Gastroenterology and Hepatology, Wroclaw Medical University, Wroclaw, Poland; ^3^Molecular Techniques Unit, Department of Forensic Medicine, Wroclaw Medical University, Wroclaw, Poland

## Abstract

**Aim:**

To assess the association of six polymorphisms in serotonin-related genes with depressive or anxiety disorders in patients with irritable bowel syndrome (IBS).

**Methods:**

The lifetime prevalence of depressive and anxiety disorders was assessed in 95 IBS patients (85% women) using the Munich version of the Composite International Diagnostic Interview (CIDI). IBS was diagnosed according to the Rome III criteria. *SCL6A4* HTTLPR polymorphism (rs4795541) was determined using PCR-based method. Single-nucleotide polymorphisms in *HTR1A* (rs6295), *HTR2A* (rs6313 and rs6311), *HTR2C* (rs6318), and *TPH1* (rs1800532) were detected by minisequencing method.

**Results:**

IBS patients with depressive disorders were characterized by higher frequency of 5-HTTLPR L allele in comparison to IBS patients with anxiety disorders. The lower frequency of 1438 A allele in *HTR2A* was found in IBS patients with depressive disorders in comparison to IBS patients without mental disorders. The lower G allele frequency in *HTR2C* rs6318 polymorphism among IBS patients with anxiety disorders was also observed.

**Conclusions:**

Our results provide further evidence for the involvement of *SLC6A4* rs4795541 and *HTR2A* rs6311 polymorphisms in the pathophysiology of depressive disorders in IBS patients. The new findings indicate that *HTR2C* rs6318 polymorphism may be associated with the susceptibility to anxiety disorders in IBS patients.

## 1. Introduction

Irritable bowel syndrome (IBS) is the most common functional gastrointestinal disorder characterized by recurrent abdominal pain accompanied by changes in bowel habits: diarrhea (IBS-D), constipation (IBS-C), or mixed bowel habits (IBS-M) [1]. IBS is also associated with a wide spectrum of extraintestinal symptoms and coexisting diseases such as mental disorders [2–4]. Epidemiological data confirm high prevalence of depressive and anxiety disorders in IBS patients ranging from 40 up to 90% of subjects [2, 5–8]. The pathophysiology of IBS is complex and not fully elucidated [1, 4]. The genetic basis of IBS has been demonstrated in family and twin studies [9, 10]. However, the results of multiple studies trying to link single-nucleotide polymorphisms (SNPs) to IBS are inconsistent [9, 11]. Serotonin-related gene variants have been extensively explored in IBS as well as in depressive and anxiety disorders [12–16]. Serotonin (5-hydroxytryptamine (5-HT)), as a main neurotransmitter of the brain-gut axis, plays a crucial role in the pathophysiology of both IBS and mental disorders [17]. Gastrointestinal dysfunction observed in IBS, as well as disturbances in the central nervous system processes, may result from alterations in 5-HT biosynthesis, release, and reuptake [17–19]. Noteworthy, serotonergic drugs are one of the most effective treatments in IBS (serotonin receptor agonists and antagonists), as well as in mood disorders (selective serotonin reuptake inhibitors) [20]. Therefore, serotonin-related genetic polymorphisms have been suggested to be associated with a high comorbidity of depressive or anxiety disorders with IBS [13, 21–24].

The aim of our study was to assess the association of polymorphisms in serotonin-related genes with depressive or anxiety disorders in IBS patients. The following six polymorphisms were included in the study: (1) the 44 bp insertion/deletion polymorphism in the promoter region (5-HTTLPR) of serotonin transporter gene (*SLC6A4*) (rs4795541), (2) the 5-HT1A receptor gene (*HTR1A*) C-1019G polymorphism (rs6295), (3) the 5-HT2A receptor gene (*HTR2A*) T102C polymorphism (rs6313), (4) *HTR2A* G-1438A polymorphism (rs6311), (5) the 5-HT2C receptor gene (*HTR2C*) cys23ser polymorphism (rs6318), and (6) the tryptophan hydroxylase-1 gene (*TPH1*) A218C polymorphism (rs1800532). The studied polymorphisms concern the key genes involved in serotonin reuptake, action, and synthesis. Their selection was based on the previous reports suggesting their role in the pathophysiology of psychiatric disorders, as well as IBS. Noteworthy, there are only few studies investigating the role of serotonin-related gene polymorphisms in IBS patients with comorbid mental disorders and they resulted in rather inconclusive findings.

## 2. Materials and Methods

### 2.1. Patients

Ninety-five patients, 81 women (85%) and 14 men (15%) admitted to the Department of Gastroenterology and Hepatology at Wroclaw Medical University (Poland), were recruited in the study. Their age ranged from 18 to 73 years (mean age: 48.8 ± 14.8 years). The study was approved by the local ethics committee (approval number KB-375/2005). A written informed consent was obtained from all patients prior to the study enrollment.

IBS was diagnosed according to the Rome III criteria (criteria fulfilled for the last 3 months with symptom onset at least 6 months before the diagnosis) [25]. In addition, based on the initial recruitment, all selected patients fulfilled also the Rome II criteria (symptom onset at least 12 months before the diagnosis). Patients with alarming symptoms or risk factors for an organic disease (such as weight loss, bleeding from the gastrointestinal tract, anemia, fever, onset of symptoms after the age of 50 years, familial occurrence of inflammatory, or neoplastic gastrointestinal diseases) were excluded from the study. In all recruited patients, colonoscopy was performed within the last five years. Subjects with cognitive impairment or serious medical conditions were not included in the study. All the patients completed a questionnaire that included demographic data, questions concerning the age of onset, frequency, and intensity of IBS symptoms and other complaints.

### 2.2. Psychiatric Diagnosis

The lifetime prevalence of depressive and anxiety disorders was diagnosed according to the ICD-10 international classification of mental disorders [26]. Each patient was examined by a senior psychiatrist using the computerized Munich version of the Composite International Diagnostic Interview (CIDI), a fully structured diagnostic instrument designed by the World Health Organization for identifying mental disorders based on diagnostic criteria of the ICD-10 and DSM-IV classification [26]. The CIDI allows to exclude psychopathological symptoms that may be a reaction to medical condition and trauma or may result from pharmacological treatment or use of alcohol and other psychoactive substances. The CIDI has been applied in many epidemiological studies in the general population as well as in studies evaluating prevalence of mental disorders among patients with various medical conditions [27, 28]. Several studies have confirmed the reliability and validity of the CIDI [29, 30].

Noteworthy, findings from the previous studies investigating the association between serotonin-related gene polymorphism and depression have suggested that the significant effect of genes observed in depressive or anxiety patients may be undetectable in case of comorbidity of these disorders [31]. Therefore, the data from the group of IBS patients with comorbid depressive and anxiety disorders were not included in the statistical analysis. IBS patients without mental disorder served as the control group in this study.

### 2.3. Genotyping

Venous blood for genotyping was taken from all patients into EDTA-containing tubes. Genomic DNA was isolated from EDTA-anticoagulated whole blood using QIAamp DNA Blood Mini Kit from QIAGEN. Allele identification of short tandem repeat (STR) polymorphism in 5-HTTLPR was performed based on polymerase chain reaction (PCR) and agarose gel electrophoresis. Single-nucleotide polymorphisms (SNPs) were detected by minisequencing method. Primers for PCR and minisequencing were designed according to DNA sequences from the NCBI dbSNP database (http://www.ncbi.nlm.nih.gov/SNP/). PCR was performed using QIAGEN Multiplex Kit. Reaction mixture contained the following components: 10 *μ*l 2x QIAGEN Multiplex PCR Master Mix, 2 *μ*l 10x mixture of primers (containing 3 *μ*M of each primer), 4 *μ*l DNA, and 4 *μ*l deionized H_2_O. PCR thermal profile consists of initial denaturation at 95°C for 15 minutes and 32 cycles: denaturation at 94°C for 30 seconds, primer annealing at 57°C for 90 seconds, and primer extension at 72°C for 90 seconds. Final incubation lasted 10 minutes at 70°C.

The analysis of PCR products was performed using electrophoresis in 1.7% agarose gel with 1x TBE buffer and ethidium bromide with the final concentration of 0.55 *μ*g/ml. Gene Ruler 100 bp DNA Ladder (Fermentas) was used for sizing and approximate quantification of amplification products. Five *μ*l of amplified fragment with the given polymorphism was purified from unincorporated dNTPs and excess primers by enzymatic method using mixture of exonuclease I (2U) and alkaline phosphatase (1U) (Fermentas). Minisequencing was performed using commercial SNaPshot Multiplex Kit (AB Applied Biosystems) according to the manufacturer's instruction.

Separation and detection of SNaPshot reaction products was performed by capillary electrophoresis using ABI PRISM 3130 Genetic Analyzer (Applied Biosystems). The results were analyzed according to the internal standard of LIZ-120 using GeneMaperID v3.2 software.

### 2.4. Statistical Analysis

Evaluation of the Hardy-Weinberg equilibrium (HWE) was performed by comparing the observed and expected genotype distribution using the *χ*^2^ test. The *χ*^2^ analysis was also used to compare categorical data between IBS patients with the diagnosis of depressive disorders, anxiety disorders, and without mental disorders. In case when observed or expected values included a cell with a value less than 5, Fisher's exact test was used. Odds ratios (OR) and 95% confidence intervals (95% CI) were calculated using the binary logistic regression model. Differences were considered as statistically significant if the *p* value was <0.05. Analyses were performed using Statistical Package for Social Sciences (SPSS) version 20.

## 3. Results

According to the bowel habit, the IBS patients were divided into three subgroups including 32% of patients with constipation (IBS-C), 32% of patients with diarrhea (IBS-D), and 36% of patients with mixed bowel habits (IBS-M) (Table 1). No statistically significant differences in the genotype distribution of the studied polymorphisms between the different IBS subtypes were found (*p* values > 0.05). An additional analysis was done in which two subgroups of the patients were compared: IBS constipated subjects versus IBS nonconstipated subjects (including both IBS-D and IBS-M) also did not reveal any significant association with the genotype distribution (*p* values > 0.05) (data not shown).

At least one depressive or anxiety disorder was diagnosed in 54 patients (57%). Depressive disorders during lifetime were diagnosed in 29 patients: in 14 (14.7%) one episode of depression, in 14 (14.7%) recurrent depressive disorder, and in one patient (1.1%) dysthymia. At least one anxiety disorder was diagnosed in 34 patients, and twelve subjects (11.6%) met the diagnostic criteria for more than one anxiety disorder. The following anxiety disorders were diagnosed: specific phobia (*n* = 24, 25.3%), general anxiety disorder (*n* = 11, 11.6%), social phobia (*n* = 10, 10.5%), agoraphobia with panic attacks (*n* = 3, 3.2%), agoraphobia without panic attacks (*n* = 4, 4.2%), other phobic anxiety disorders (*n* = 10, 10.5%), and panic disorder (*n* = 3, 3.2%). According to the diagnosis based on the CIDI examination, all IBS patients were divided into four groups: without diagnosis of depressive nor anxiety disorder (*n* = 41, 43%), with depressive disorders only (*n* = 20, 21%), with anxiety disorders only (*n* = 25, 26%), and with comorbid depressive and anxiety disorders (*n* = 9, 10%).

The allele frequency and genotype distribution of the analyzed genes in three subgroups are presented in Table 2. Due to the technical reasons in one IBS patient, 2 genotypes were not evaluated. The genotype distribution for all polymorphisms was consistent with the Hardy-Weinberg equilibrium.

### 3.1. SLC6A4

There were statistically significant differences in the distribution of 5-HTTLPR genotypes (*χ*^2^ = 6.19, df = 2, *p* = 0.04) between patients with depressive disorders and anxiety disorders. IBS patients with depressive disorders were characterized by higher frequencies of 5-HTTLPR L allele in comparison to IBS patients with anxiety disorders (OR = 0.34, 95% CI = 0.13–0.86, *p* = 0.02). There were no statistically significant differences in the distribution of 5-HTTLPR genotypes between IBS patients with coexisting anxiety disorders and patients with no mental disorders or between IBS patients with coexisting depressive disorder and patients with no mental disorders.

### 3.2. HTR1A

Among IBS patients with depressive disorders comparing to IBS patients with anxiety disorders, the homozygous G/G genotype of *HTR1A* C-1019G polymorphism occurred less frequently (0.15 versus 0.36) and the difference was also observed between individuals with depressive disorders and without mental disorders (0.15 versus 0.32). The C allele occurred with a higher frequency in IBS patients with depressive disorders (0.60) than in the patients either with anxiety disorders (0.46) or without mental disorders (0.44). However, the differences in the frequency of alleles and genotypes of *HTR1A* C-1019G polymorphism between all compared groups (anxiety group versus no mental disorder group, depression group versus no mental disorder group, and anxiety group versus depression group) were not statistically significant (for alleles *p* = 0.80, *p* = 0.09, and *p* = 0.19, resp., and for genotypes *p* = 0.62, *p* = 0.25, and *p* = 0.28, resp.).

### 3.3. HTR2A

No statistically significant differences were found between the compared groups in the frequency of alleles or genotypes of *HTR2A* T102C polymorphism (data not shown). On the other hand, the analysis of the *HTR2A* G-1438A polymorphism revealed the trend level significant difference in the frequency of alleles between patients with depressive disorders and without mental disorders (OR = 2.01, 95% CI = 0.93–4.35, df = 1, *p* = 0.07). Subjects with depressive disorders were more likely to have G allele than A allele (0.60 versus 0.40), although there were no significant differences in the distribution of genotypes between IBS patients with depressive disorders and IBS patients without mental disorders (*χ*^2^ = 3.12, df = 2, *p* = 0.21). There were no significant differences in the distribution of genotypes between IBS patients with anxiety disorders and IBS patients without mental disorders (*χ*^2^ = 1.41, df = 2, *p* = 0.49) and between IBS patients with coexisting depressive disorders and IBS patients without mental disorders (*χ*^2^ = 3.12, df = 2, *p* = 0.38).

### 3.4. HTR2C

The analysis of *HTR2C* cys23ser polymorphism revealed that among IBS patients with anxiety disorders, G allele (that encodes the 23cys variant) was less common than C (that encodes the 23ser variant) (0.74) comparing to patients with depressive disorders (0.82) and without mental disorders (0.89). The difference in the allele frequency between patients with anxiety disorders and patients without mental disorders was statistically significant (OR = 0.35, 95% CI = 0.14–0.90, *p* = 0.02). There were trend level differences in the distribution of genotypes in *HTR2C* cys23ser polymorphism between IBS patients with comorbid anxiety disorders and IBS patients with no mental disorders (*χ*^2^ = 5.68, df = 2, *p* = 0.06).

### 3.5. TPH1

With respect to *THP1* A218C polymorphism, no statistically significant differences between the compared groups were found (data not shown).

## 4. Discussion

In the current study, six different polymorphisms of five serotonin-related genes in the group of IBS patients with comorbid depressive or anxiety disorders diagnosed according to the diagnostic criteria of ICD-10 were investigated.

### 4.1. SLC6A4

*SLC6A4* encoding the 5-HT transporter protein occurs in some polymorphic variants among which the most extensively studied, both in the psychiatric field and IBS, is 44-bp insertion/deletion polymorphism in the promoter region (5-HTTLPR) [12–16]. It results in the short (S) and long (L) alleles which are associated with the different transcriptional activity and protein expression level leading to changes in serotonin reuptake [32]. Homozygous L/L genotype determines higher reuptake of serotonin from synapses than homozygous S/S genotype or heterozygous L/S genotype [33]. A number of prior studies showed considerable variability of genotype distribution in different populations, but most of them did not find any significant difference in the distribution of 5-HTTLPR polymorphism between the healthy individuals and IBS patients [34, 35]. However, some findings suggested the association between this *SLC6A4* polymorphism and different clinical subtypes of IBS [36–43]. Several meta-analyses revealed conflicting results reporting on no association with *SLC6A4* polymorphism and IBS subtypes [44] or IBS overall [45], as well as finding a positive association between *SLC6A4* polymorphism and IBS-C [46]. The most recent meta-analysis with the largest sample size, which included 25 studies involving 3443 IBS patients and 3359 controls, found a positive association between L allele and L/L genotype and IBS-C only in the East Asian population, but not in the Caucasian population [47]. The reported ethnic difference could result from the significantly lower frequency of L allele among the East Asian population in comparison to Caucasian controls, as evidenced by the meta-analysis [47].

In our study, no statistically significant differences in the genotype distribution of the studied polymorphisms between the different IBS subtypes were found. Due to the small sample size, we did not analyze the association of *SLC6A4* polymorphism with different mental disorders in three different subtypes of IBS.

Numerous studies have suggested the relationship between *SLC6A4* polymorphisms and some mental disorders, including depressive and anxiety disorders [12, 13, 33, 48–50]. In our study, the frequency of 5-HTTLPR alleles, as well as genotype distribution, significantly differed between IBS patients with anxiety disorders and IBS patients with depressive disorders. Interestingly, in both groups, the most common allele was L allele; however, among patients with anxiety disorders, the difference in allele frequency was much lower than that among depressive patients. In the group with anxiety disorders, the most frequent genotype was heterozygous L/S genotype, while patients with depressive disorders were more likely to have homozygous L/L genotype and no S/S genotype was found in this group. To the best of our knowledge, only one other study investigated the association of this *SLC6A4* polymorphism with depressive and anxiety disorders in IBS (not including a few studies assessing only depressive or anxiety symptoms, not disorders). Jarrett et al. [13] using the CIDI to diagnose depressive and anxiety disorders during lifetime analyzed two polymorphisms of *SLC6A4* in the group of 138 patients with IBS (mostly women—85%, similar as in our study). Contrary to our findings, Jarrett et al. [13] found that patients with homozygous S/S genotype were more likely to have diagnosis of depressive disorders (recurrent depression) during lifetime. They did not reveal any relationship between alleles or genotypes and anxiety disorders, but interestingly reported on the association between L/L genotype and decrease in social functioning in IBS [13]. However, Jarrett et al. [13] did not report on comorbidity of depressive and anxiety disorders in IBS patients. In our group, comorbid depressive and anxiety disorders were diagnosed in 10% of the subjects. Previous reports have indicated that a coexistence of more than one mental disorder could affect the results of genetic studies. For example, Rotondo et al. [51] presented evidences referring to the association study of *SLC6A* and bipolar disorders. In order to have homogenous groups, we excluded IBS patients with comorbid depressive and anxiety disorders from the analysis.

### 4.2. HTR1A

No significant differences in the frequency of alleles and genotypes of *HTR1A* C-1019G polymorphism were found. However, the IBS patients with depressive disorders were less likely to have homozygous G/G genotype comparing to the patients with anxiety disorders or without mental disorders. *HTR1A* C-1019G polymorphism has been widely investigated as the 5-HT1A receptor has been considered to be strongly involved in the pathophysiology of depressive and anxiety disorders [14]. However, the results of previous studies are contradictory. Lemonde et al. [52] found a strong relationship between G allele and major depression, whereas Arias et al. [53] and Huang et al. [14] did not observe any differences in the G allele frequency between the group with major depression and controls.

### 4.3. HTR2A

Pata et al. [54] reported on a high occurrence of C allele of *HTR2A* T102C polymorphism in the group of 55 IBS patients. Moreover, the homozygous C/C genotype was related to a higher risk of anxiety disorders than other genotypes. In contrast, we did not find any difference in the frequency of alleles and genotypes comparing the groups with anxiety disorders and without mental disorders and similarly comparing the groups with anxiety disorders and with depressive disorders [55]. Noteworthy, in the study of Pata et al. [54], the Hospital Anxiety and Depression Scale (HADS) was used to diagnose depression and anxiety among IBS patients. However, HADS is a screening tool that allows to assess only the probability of mental disorder that requires further examination. The findings of Pata et al. [54] suggesting the associations of *HTR2A* T102C polymorphism with IBS have not been replicated. In a more recent study, the differences in the genotype distribution in *HTR2A* polymorphism between healthy individuals and IBS patients have not been found [56].

Markoutsaki et al. [56], as well as Pata et al. [54], indicated that the A allele carriers of *HTR2A* G-1438A polymorphism might have a higher risk of IBS comparing to the G allele carriers. In our study, IBS patients with depressive disorders were characterized by the lower frequency of A allele in *HTR2A* G-1438A polymorphism in comparison to IBS patients without any mental disorder. We did not observe any difference in the frequency of A allele among IBS patients with anxiety disorders comparing to the subjects without mental disorders.

### 4.4. HTR2C

In our study, anxiety disorders in IBS patients were related to the higher frequency of C allele and C/C genotypes of *HTR2C* cys23ser polymorphism comparing to IBS patients without mental disorders. The difference was statistically significant. Previous studies suggested the role of *HTR2C* in the pathogenesis of some mental disorders and pathological behaviours [57–60]; however, no study has investigated the role of *HTR2C* polymorphism in the pathophysiology of IBS or its association with IBS and mental disorder comorbidity so far.

What may be interesting in the context of IBS and mental disorders is the fact that the 5-HT2C receptor is considered to be involved in stress response, anxiety, and pain modulation [57]. However, only few studies investigated the association of *HTR2C* cys23ser polymorphism and anxiety disorders resulting in the conflicting findings. Fehr et al. [61] found that *HT2C* cys23ser allele and genotype frequencies did not differ among patients with panic disorder nor generalized anxiety disorder comparing to healthy subjects. On the other hand, Pritchard et al. [62] reported an increase in C allele and C/C genotype frequencies of *HTR2C* cys23ser polymorphism among females with anxiety in the course of Alzheimer's disease. Mickey et al. [63] found greater dopamine release among healthy individuals with C alleles (23ser variant) in reaction to deep muscular pain. According to the authors, *HTR2C* cys23ser polymorphism may play a key role in the pathophysiology of stress-related diseases, and it may increase the risk of some neuropsychiatric disorders due to greater stress-induced dopamine release in mesoaccumbal dopaminergic circuitry [63].

The C allele of *HTR2C* that encodes 23ser variant is also suggested to be associated with a higher risk of recurrent major depression and bipolar disorder, but not all the findings support this report [64, 65]. In our study, we did not find a difference between IBS patients with depressive disorders and without mental disorders in the frequency of alleles or genotypes. In spite of the contradictory findings concerning the involvement of *HTR2*C cys23ser polymorphism in the pathogenesis of mental disorders, the researchers agree that it may affect clinical course or may be related with the onset of affective disorders [66].

### 4.5. TPH1

Tryptophan hydroxylase (TPH) is the rate-limiting enzyme of serotonin synthesis. TPH1 is one of two isoforms that is found both in the brain and in the gut [17]. Polymorphisms of the TPH1 gene (*TPH1*) may result in dysfunction of the serotonin system; thus, in many studies, the association of *TPH1* polymorphisms and some mental (mostly affective) disorders has been investigated [15, 16].

Chen et al. [16] included in their meta-analysis 27 studies concerning bipolar and major depressive disorders. They found that the A/A genotype of *TPH1* A218C polymorphism was associated with the risk of bipolar disorder in the Caucasian population. They did not find an association between this polymorphism and major depression [16].

*TPH1* is considered to play an important role in the regulation of serotonin activity, not only in the central nervous system but also in the gastrointestinal tract as well. Therefore, some studies focused on the association of its polymorphisms with IBS [67]. However, only one study involved IBS patients with mental disorders [68]. Jun et al. [68] failed to find any association between *TPH* polymorphisms and lifetime history of mental health disorders measured by the CIDI in women with IBS. Likewise, we did not find any difference in allele and genotype frequency between patients with depressive and anxiety disorders and without mental disorders.

### 4.6. Strengths and Limitations of the Study

The aim of this study was to evaluate the association of serotonin-related gene polymorphisms with psychiatric comorbidity in IBS patients. Firstly, the cardinal value of the study is based on the fact that all patients were properly diagnosed by psychiatrists using the CIDI. It is important to emphasize that HADS, commonly used in some other studies, allows only to assess the presence and severity of anxiety and depressive symptoms, while the proper diagnosis of mental disorder requires further verification. Secondly, the number of assessed associations in this study is relatively high as six different polymorphisms were evaluated.

Since the aim of the study was to perform the comparison between IBS patients subgroups divided according to the comorbidity of mental disorders, as the control group served IBS patients without any mental disorders. IBS subjects with concomitant depressive and anxiety disorders were excluded from the analysis. We did not find any statistically significant differences in the genotype distribution of the studied polymorphisms between the different IBS subtypes according to the predominant bowel habit. Due to the small sample size, we did not analyze differences regarding psychiatric comorbidity in different IBS subtypes.

The female predominance in the studied group constitutes one of the limitations of the study. It may potentially affect the results due to gender-dependent differences in genotype distribution. For example, Niesler et al. [69] reported low frequency of SS genotype in 5-HTTLPR in male patients with IBS. Many discrepancies between the studies may be also related to the ethnic differences in genotype distribution as well as to the heterogeneity of IBS patient group. Psychiatric comorbidity itself constitutes another confounding factor. Additionally, the effect of overlap of more than one coexisting mental disorders should be considered [31].

Noteworthy, all IBS patients were recruited in the tertiary care center. It may result in a high percentage of psychiatric comorbidity in the studied group. Additionally, these subjects, even if without mental disorders, may represent more severe IBS cases.

Finally, this study was performed in the frame of gene specific candidate driven approach, while there is a growing body of evidence that genome-wide association studies (GWAS) may present a valuable alternative to discover genetic risk loci in such a complex disease as IBS [10, 11]. Furthermore, more comprehensive approach including gene-environment interaction and epigenetic studies are also needed [9].

In conclusion, there are only few studies investigating the role of serotonin-related gene polymorphisms in the comorbidity of IBS and mental disorders. The findings of the present study indicate that comorbid depressive or anxiety disorders may affect the results of genetic studies in IBS patients. Our results provide further evidence for the involvement of *SLC6A4* rs4795541 and *HTR2A* rs6311 polymorphisms in the pathophysiology of depressive disorders in IBS patients. The new findings indicate also that *HTR2C* rs6318 polymorphism may be associated with the susceptibility to anxiety disorders in IBS patients. Certainly, due to the small sample size and other limitations of the study, as well as the heterogeneous and multifactorial character of IBS, the reported findings should be interpreted with caution and need further validation and replication.

## Figures and Tables

**Table 1:** Characteristics of the studied IBS patients.

IBS subtype	Number of patients (%)	Female patients (*n*)	Male patients (*n*)	Patients without mental disorders (*n*)	Patients with depressive disorders (*n*)	Patients with anxiety disorders (*n*)	Patients with comorbid anxiety and depressive disorders (*n*)
IBS-C	30 (32%)	28	2	12	5	8	5
IBS-D	30 (32%)	23	7	17	4	8	1
IBS-M	35 (36%)	30	5	12	11	9	3

**Table 2:** Frequency of alleles and genotypes in IBS patients without mental disorders and with depressive or anxiety disorders.

Gene	SNP database number (RS)	Functional variant	Alleles and genotypes		Patients without mental disorders *n* (frequency)	Patients with depressive disorders *n* (frequency)	Patients with anxiety disorders *n* (frequency)
*SLC6A4*	rs4795541	c.-1950_-1949insC	Alleles	L	53 (0.62)	31 (0.77)	27 (0.54)
				S	29 (0.38)	9 (0.23)	23 (0.46)
			Genotypes	L/L	19 (0.46)	11 (0.55)	8 (0.32)
				L/S	15 (0.37)	9 (0.45)	11 (0.44)
				S/S	7 (0.17)	0	6 (0.24)
				HWE	*p* = 0.12	*p* = 0.19	*p* = 0.65
							
*HTR1A*	rs6295	c.1019C>G	Alleles	C	35 (0.44)	24 (0.60)	23 (0.46)
				G	45 (0.56)	16 (0.40)	27 (0.54)
			Genotypes	C/C	8 (0.20)	7 (0.35)	7 (0.28)
				C/G	19 (0.48)	10 (0.50)	9 (0.36)
				G/G	13 (0.32)	3 (0.15)	9 (0.36)
				HWE	*p* = 0.83	*p* = 0.85	*p* = 0.17
							
*HTR2A*	rs6313	c.102C>T	Alleles	C	47 (0.59)	22 (0.55)	28 (0.56)
				T	33 (0.41)	18 (0.48)	22 (0.44)
			Genotypes	C/C	13 (0.32)	5 (0.25)	9 (0.36)
				C/T	21 (0.53)	12 (0.60)	10 (0.40)
				T/T	6 (0.15)	3 (0.15)	6 (0.24)
				HWE	*p* = 0.34	*p* = 0.60	*p* = 0.35
	rs6311	c.-1438G>A	Alleles	A	47 (0.57)	16 (0.40)	25 (0.50)
				G	35 (0.43)	24 (0.60)	25 (0.50)
			Genotypes	A/A	14 (0.34)	3 (0.15)	8 (0.32)
				A/G	19 (0.46)	10 (0.50)	9 (0.36)
				G/G	8 (0.20)	7 (0.35)	8 (0.32)
				HWE	*p* = 0.54	*p* = 0.65	*p* = 0.16
							
*HTR2C*	rs6318	c.68C>G	Alleles	C	9 (0.11)	7 (0.18)	13 (0.26)
				G	73 (0.89)	33 (0.82)	37 (0.74)
			Genotypes	C/C	0	0	1 (0.04)
				C/G	9 (0.22)	7 (0.35)	11 (0.44)
				G/G	32 (0.78)	13 (0.65)	13 (0.52)
				HWE	*p* = 0.37	*p* = 0.43	*p* = 0.34
							
*TPH1*	rs1800532	c.218A>C	Alleles	A	36 (0.44)	19 (0.47)	19 (0.38)
				C	46 (0.56)	21 (0.53)	31 (0.62)
			Genotypes	A/A	10 (0.24)	3 (0.15)	5 (0.20)
				A/C	16 (0.39)	13 (0.65)	9 (0.36)
				C/C	15 (0.37)	4 (0.20)	11 (0.44)
				HWE	*p* = 0.11	*p* = 0.17	*p* = 0.24

HWE: Hardy-Weinberg equilibrium test.
